# Zebrafish EEG predicts the efficacy of antiepileptic drugs

**DOI:** 10.3389/fphar.2022.1055424

**Published:** 2022-12-08

**Authors:** Jun-Nyeong Shin, Ki-Baek Lee, Woojae Butterworth, Soo-Kyung Park, Jung-Yeon Kim, Sohee Kim

**Affiliations:** ^1^ Department of Robotics and Mechatronics Engineering, Daegu Gyeongbuk Institute of Science and Technology (DGIST), Daegu, South Korea; ^2^ Zefit Inc, Daegu, South Korea; ^3^ School of Undergraduate Studies, Daegu Gyeongbuk Institute of Science and Technology (DGIST), Daegu, South Korea

**Keywords:** zebrafish, electroencephalogram (EEG), drug screening, antiepileptic drug (AED), efficacy

## Abstract

**Background:** Pharmacological evaluation of antiepileptic drugs (AEDs) using mammalian animals takes long time and is expensive. The zebrafish is a species commonly used to study brain functions, neurological diseases, and drug toxicity, and attracts more attention as an alternative animal model to substitute or supplement mammalian animals in drug development. Electroencephalogram (EEG) is a key indicator for diagnosing brain diseases such as epilepsy, by directly measuring the brain activity. We propose a novel method for pharmacological evaluation of AEDs based on EEG from adult zebrafish, which allows researchers to select more clinically valuable drugs at the early stage of AED screening.

**Methods:** To evaluate the efficacy of AEDs, zebrafish EEG signals were measured after administering six AEDs (valproate acid, gabapentin, ethosuximide, oxcarbazepine, tiagabine, and topiramate) at various doses to pentylenetetrazol (PTZ)-induced seizure models. The change in seizure activity was investigated according to doses. The antiepileptic effect was determined by observing a significant decrease in at least one out of three indicators of the number, total duration, and mean duration of ictal events.

**Results:** Using EEG signals from adult zebrafish, antiepileptic effects were observed with all six AEDs. Among them, antiepileptic effects depending on dose were confirmed with valproate acid, gabapentin, ethosuximide, and tiagabine. Moreover, the 50% effective doses (ED50) of valproate acid and tiagabine were determined based on zebrafish EEG for the first time, indicating that the quantitative inter-species comparison of the AED efficacy is possible between zebrafish and mammals such as rodents.

**Significance:** The results show that zebrafish can be used to effectively and quantitatively evaluate the efficacy of AEDs based on EEG, the same method to evaluate antiepileptic effects in mammals, suggesting that the proposed method can contribute in reducing the cost and duration of search for AEDs and thus accelerate the drug development cycles.

## Introduction

Epilepsy is a common neurological disorder with a variety of underlying etiologies, and seizures are its main symptom, which affects 1%–2% of the world’s population (McNamara, 1999). Patients with epilepsy suffer from unpredictable seizures, resulting in high mortality, comorbidities, and high medical-care costs. Many successful antiepileptic drugs (AEDs) have been developed over more than 100 years of research; however, 30% of patients with drug-resistant epilepsy cannot be treated using conventional AEDs (Sillanpää and Schmidt, 2006), and expensive healthcare is not accessible in low- and middle-income countries. Therefore, low-cost and new AEDs are urgently needed.

The zebrafish (Danio rerio) is an animal species commonly used to study and model brain functions and disorders. Compared to other animal models, zebrafish have the advantages of a low cost of maintenance and easy handling (Grone and Baraban, 2015). They are also useful genetic models due to their short generation durations and high fertility (Cho et al., 2015). Moreover, zebrafish can be used to verify the efficacy of treatment methods at the complex organ or even organism levels. Unlike fruit flies (Drosophila), zebarfish are vertebrates, and share 75% of disease genes with humans (Dümpelmann et al., 2015; Heischmann et al., 2016). The characteristics of epileptic seizures in zebrafish are known to be similar to those of humans and mice (Zijlmans et al., 2012; Afrikanova et al., 2013; Cho et al., 2017).

Electroencephalogram (EEG) is the electrophysiological signals that directly exhibit changes in neuronal (and neuronal network) activity. In clinical practice, EEG is commonly used to diagnose epilepsy, and seizures are observed through ictal event signals (Zijlmans et al., 2012; Dümpelmann et al., 2015; Heischmann et al., 2016). Therefore, in many studies, EEG has been used as a key indicator to verify the efficacy of epilepsy treatment.

In most cases, the effectiveness of AEDs in zebrafish epilepsy models has been evaluated through behavioral analysis, mainly because of the easy implementation of behavioral analysis and the availability of mass screening. Automated video tracking software is used to detect the total distance moved and the speed of movement. Compared to the control groups, the epilepsy model groups travel a longer distance and move more quickly; thus, these variables can be used to evaluate the efficacy of a drug (Afrikanova et al., 2013). Although large-scale screening of the effectiveness of AEDs can be conducted *via* behavioral analysis, it has several limitations; for example, drugs such as sedatives and muscle relaxants can produce false-positive results. In addition, all such behavioral analyses have been performed on larvae, and a systematic method for evaluating the efficacy of AEDs in adult zebrafish is lacking. Zebrafish, unlike other organisms, show a clear larval-adult duality. Larvae have fewer organs and less developed nervous and endocrine systems; thus, drugs may have different effects on larvae and adults (Cho et al., 2017).

Ictal events can be observed from the EEG signals of zebrafish epilepsy models. In addition, the effectiveness of an anticonvulsant drug can be verified by detecting ictal events, which serve as the key indicator and are represented by the number, total duration, and mean duration of ictal events (Griffin et al., 2017). According to a previous study (Griffin et al., 2018), EEG-based evaluations of AED efficacy are not susceptible to the false-positive limitations that are observed through behavior-based evaluations of AED efficacy. Generally, screening AEDs to determine their efficacy is possible in both adults and larvae (Baraban et al., 2013; Cho et al., 2017). In particular, adult zebrafish have a mature central nervous system, and enable the oral or intraperitoneal administration of drugs, which permits the accurate drug delivery as well as the administration of insoluble drugs, and thereby pharmacological comparisons with results in mammals (Cho et al., 2020). However, it has been difficult to measure the brain waves of multiple adult zebrafish simultaneously due to the difficulty of achieving precise electrode localization. Although studies on screening the efficacy of several AEDs using EEG have been conducted (Baraban et al., 2013; Eimon et al., 2018), none of these studies have used adult zebrafish. Recently, a system that measures the brain waves of multiple adult zebrafish in parallel was developed, suggesting the possibility of large-scale drug screening on adults (Lee et al., 2020).

In the present study, we propose a method that quantitatively evaluates the antiseizure effect of various AEDs based on this previously developed EEG recording system using adult zebrafish. To validate the use of EEG data as a quantitative indicator of the efficacy of AEDs in drug screening applications, six clinically used AEDs were administered at various doses to a representative epilepsy model, and the corresponding EEG signals were recorded. The specific ictal events were quantitatively analyzed from the EEG, and the efficacy of AEDs at different doses was analyzed.

The method used to evaluate the efficacy of AEDs in this study is capable of assessing the effects of multiple drugs under the same environmental conditions through a rapid efficacy analysis, enabling more reliable comparisons between drugs and studies. In addition, this method provides an economical and accurate way to quantitatively evaluate drugs through an EEG analysis in zebrafish. The proposed method can contribute to rapid decision-making during drug development by enabling the pharmacological efficacy of a treatment to be confirmed at early stages.

## Materials and methods

### Animals

Adult wild-type zebrafish were provided by the Zebrafish Center for Disease Modeling, Korea, and maintained in a standard zebrafish facility at Daegu Gyeongbuk Institute of Science and Technology (DGIST) with a 14 h light:10 h dark cycle. Water in the tank was kept under mechanical and chemical filtration at a targeted temperature of 28 ± 0.5°C and pH of 7.0–8.0. We fed Brine shrimp and flake food (Tetramin, Germany) to zebrafish twice a day. After each recording session, individual animals were euthanatized by rapid chilling with cold water at temperatures around 2°C–4°C (Grush et al., 2004; Matthews and Varga, 2011). We used a total of 528 males to investigate the effects of AEDs on EEG signals. All experimental procedures were approved by the Institutional Animal Care and Use Committee of DGIST.

### Chemicals

We used 15 ppm and 7.5 ppm eugenol (Merck, Germany) for initial anesthesia and sustained anesthesia of the zebrafish, respectively, similar to the methods used by other studies (Pineda et al., 2011; Collymore et al., 2014; Cho et al., 2017). Additionally, we orally administered 220 mg/kg pentylenetetrazole (PTZ) (Merck, Germany), the convulsant, while keeping the fish anesthetized. Based on the zebrafish body weight of 200 mg, 4 µl of AED solution was administered. Stage 3 anaesthesia was induced by placing zebrafish in eugenol solution for 5 min before EEG measurement (Cho et al., 2017). During EEG measurement, 7.5 ppm of eugenol solution was continuously supplied orally. The reason why we chose oral administration among various drug administration methods, such as intraperitoneal or intravenous administration, was that oral administration can reduce traumatic stress to animals. Also, the oral administration taking body weight into account is considered as a more controlled drug delivery method than exposure by immersion, enabling the experimenter to know the precise dose applied to animals.

All AEDs used in this study were selected from AEDs that are commonly used clinically: valproate acid (VPA), gabapentin (GBP), ethosuximide (ETS), oxcarbazepine (OXC), tiagabine (TGB), and topiramate (TOP) (Merck, Germany). To investigate the difference in efficacy according to the evaluation methods, we chose three drugs (VPA, GBP, TOP) that had shown the efficacy in a previous zebrafish EEG study (Afrikanova et al., 2013) and three other drugs (ETS, OXC, TGB) with which the efficacy was invisible using zebrafish EEG (Afrikanova et al., 2013). The dosages of each AED were determined based on the literature, as shown in Supplementary Table S1. Each group treated with an AED was cotreated with PTZ before EEG measurement. All drugs were dissolved in ultrapure water. DMSO 0.1% was used to dissolve VPA, GBP, ETS, OXC and TGB, while DMSO 10% was used to dissolve TOP.

### Electrodes and the EEG recording system

We collected EEGs using a dedicated EEG recording system for adult zebrafish, as shown in Supplementary Figure S1. The recording electrode was a copper core electrode with a diameter of 1 mm. The EEG recording system was connected to a biosignal acquisition system (Biopac, USA), and EEG signals were measured simultaneously from 8 animals. To record the zebrafish EEG out of the water, EEGs were recorded as illustrated in Supplementary Figure S1. Each zebrafish was held in a fish trap with dimensions of 20 mm × 110 mm × 15 mm. The fish were fixed in a space that was a maximum of 6.85 mm × 38 mm. Eugenol was administered into the mouth of the trapped zebrafish using a needle to maintain anesthesia. The fish trap had a structure that allowed it to drain and thus keep the head of the fish dry while administering eugenol. EEG data were recorded by attaching the electrode to the zebrafish head using a precision X-, Y- and Z-axis manipulator and a camera for visual guidance. To reduce external noises as much as possible, EEG data were recorded inside a shielded chamber, which was made of steel and designed to block electromagnetic interferences and sounds (Sontek, Korea). The dimensions of the inside of the shielded chamber were 120 cm × 60 cm × 80 cm.

### Data analysis

To eliminate electrical noise at 60 Hz, we used a 0.5-55 Hz bandpass filter provided by Biopac software, and recorded the EEG at a sampling rate of 10 kSa/s. EEG data recorded using Biopac Student Lab 4.1 were saved on a laptop. The power spectral density (PSD) of EEG signals was obtained by Fourier analysis, which decomposes EEG signals in the time domain into frequency components (Borbély et al., 1981). Data were analyzed by dividing the bandwidth into five bands (Amzica and Steriade, 1998; Baumeister et al., 2008): the delta (0.5 Hz–4 Hz), theta (4 Hz–8 Hz), alpha (8 Hz–13 Hz), beta (13 Hz–30 Hz), and gamma bands (30 Hz–55 Hz). Analysis of EEG signals was performed using MATLAB software (MathWorks, Inc., USA).

Statistical analysis was performed based on one-way analysis of variance (ANOVA), followed by Dunnett’s test (*post hoc*) using Prism 9 software (GraphPad Software Inc., USA). Therefore, all adjusted *p*-values were the result of Dunnett’s test. When comparing between groups, statistical significance was determined to be present if *p* < 0.05. F values were used for ANOVA, which represent the ratio of the explained variance to the unexplained variance. A value of F = 1 indicates that the two variances are equal. A large F-value means that the between-group variation is greater than the within-group variation, which can be interpreted that there is a statistically significant difference in the group means.

The 50% effective doses (ED50) curve represents the drug efficacy when the number, total duration, or mean duration of ictal events for each individual in a drug-treated group was 50% or less than the average values of the three indicators of the PTZ-only group. The ED50 value was calculated using the proportion of samples with a positive efficacy in the group.

## Results and discussion

### Ictal events are clearly observed in EEG signals from PTZ-induced zebrafish epilepsy models

**FIGURE 1:**
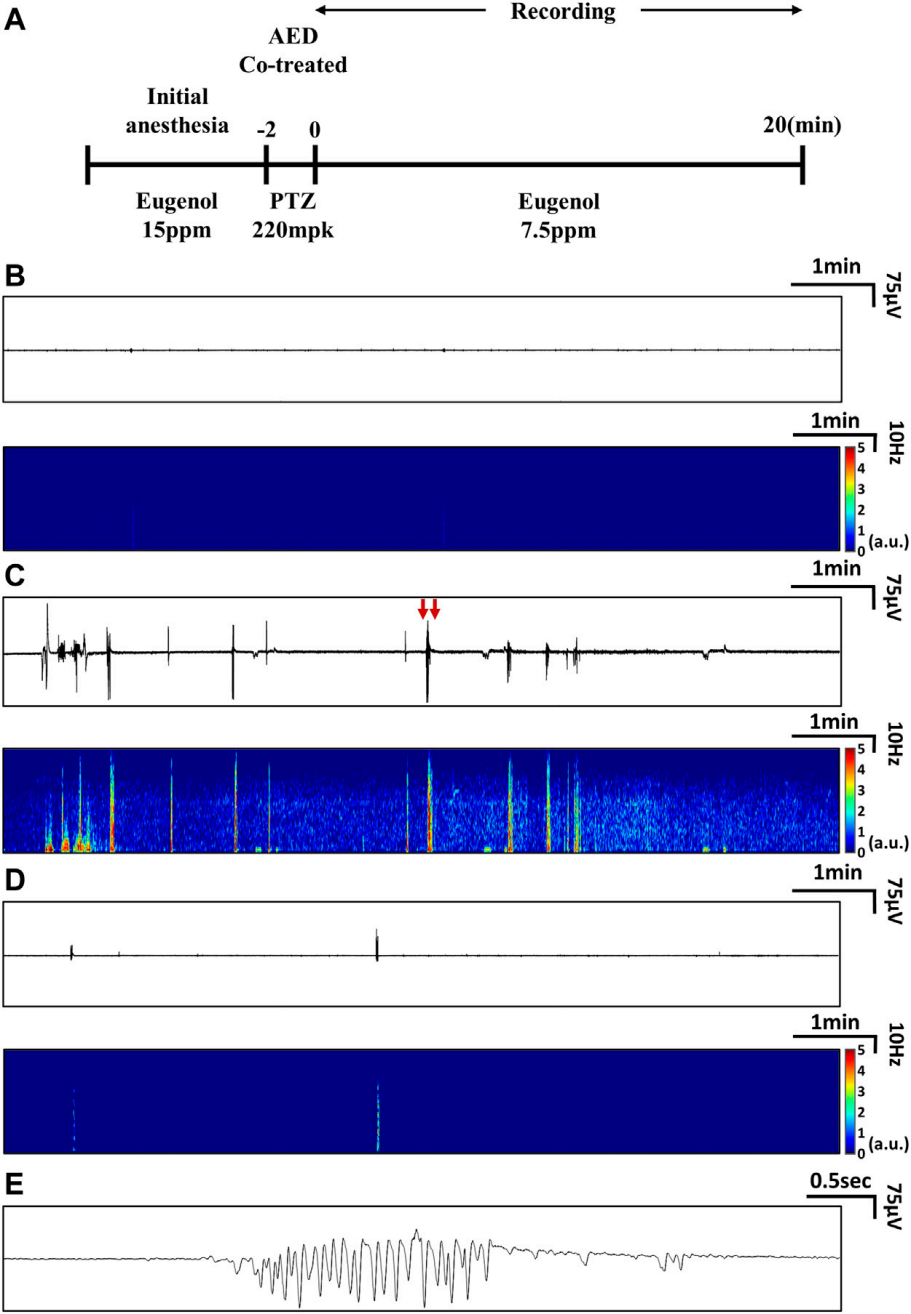
Experimental protocols used to measure EEG signals and representative EEG data. **(A)** Protocols used to measure EEG signals from the PTZ-induced seizure model treated with AEDs. **(B-E)** Examples of real-time EEG data and time-frequency plots: **(B)** EEG from a zebrafish that received neither PTZ nor AED treatment (control), **(C)** EEG from a zebrafish treated with PTZ only, and **(D)** EEG from a zebrafish cotreated with PTZ and VPA. **(E)** Magnified view of ictal signals indicated by red arrows in **(C)**.

We measured EEG signals according to the protocol presented in Figure 1A. Three experimental groups were used: a group treated with vehicle (E3 medium + DMSO 0.1% or 10%) that served as the control, a group treated with PTZ only, and a group cotreated with PTZ and VPA simultaneously. Eight male zebrafish were included in each group. In all groups, EEG signals were recorded for 20 min with one channel per zebrafish. The control group showed no ictal events and peak-to-peak amplitudes of 0.05 mV on average, as shown in Figure 1B.

In the PTZ-only group, ictal signals were observed, as shown in Figure 1C. Signals with peak-to-peak amplitudes of approximately 0.3 mV were observed during ictal periods after PTZ administration, and the number and magnitude of ictal events decreased over time. During the ictal periods, an increase in signal amplitude was observed in all frequency domains (0.5∼55 Hz), as shown in Figure 1C. In the group cotreated with PTZ and VPA, ictal events were observed, as shown in Figure 1D, but compared to the PTZ-only group, these ictal events were clearly suppressed. As shown in Figure 1E, the waveform of ictal signals was confirmed and characterized by relatively periodic and large-scale signals compared to those of the control group.

The tonic-clonic-like seizures observed in the PTZ model had high signal amplitudes at all frequencies, especially at frequencies of 5–7 Hz. Absence-like seizures in PTZ models are characterized by high signal amplitudes at all frequencies, especially at frequencies of 2–3 Hz (Cho et al., 2017). Thus, these two types of ictal events could be distinguished, as shown in Supplementary Figure S2. Additionally, as shown in Supplementary Table S2, the EEG patterns of epileptic zebrafish, mice, and humans are similar. The characteristics of ictal events observed in the zebrafish EEG signals, as shown in Supplementary Figure S3, are similar to tonic-clonic-like and absence-like seizure patterns described in mouse models (Hitt et al., 2010).

### EEG signals clearly show that ictal events are suppressed by various AEDs

We recorded EEG signals after administering various AEDs along with PTZ. The AEDs used were VPA, GBP, ETS, OXC, TGB, and TOP (Merck, Germany). All AEDs were cotreated with PTZ (Hwang et al., 2020). Each of these six groups contained eight animals. The drugs were administered orally as described in a previous study (Nieoczym et al., 2012).

**FIGURE 2:**
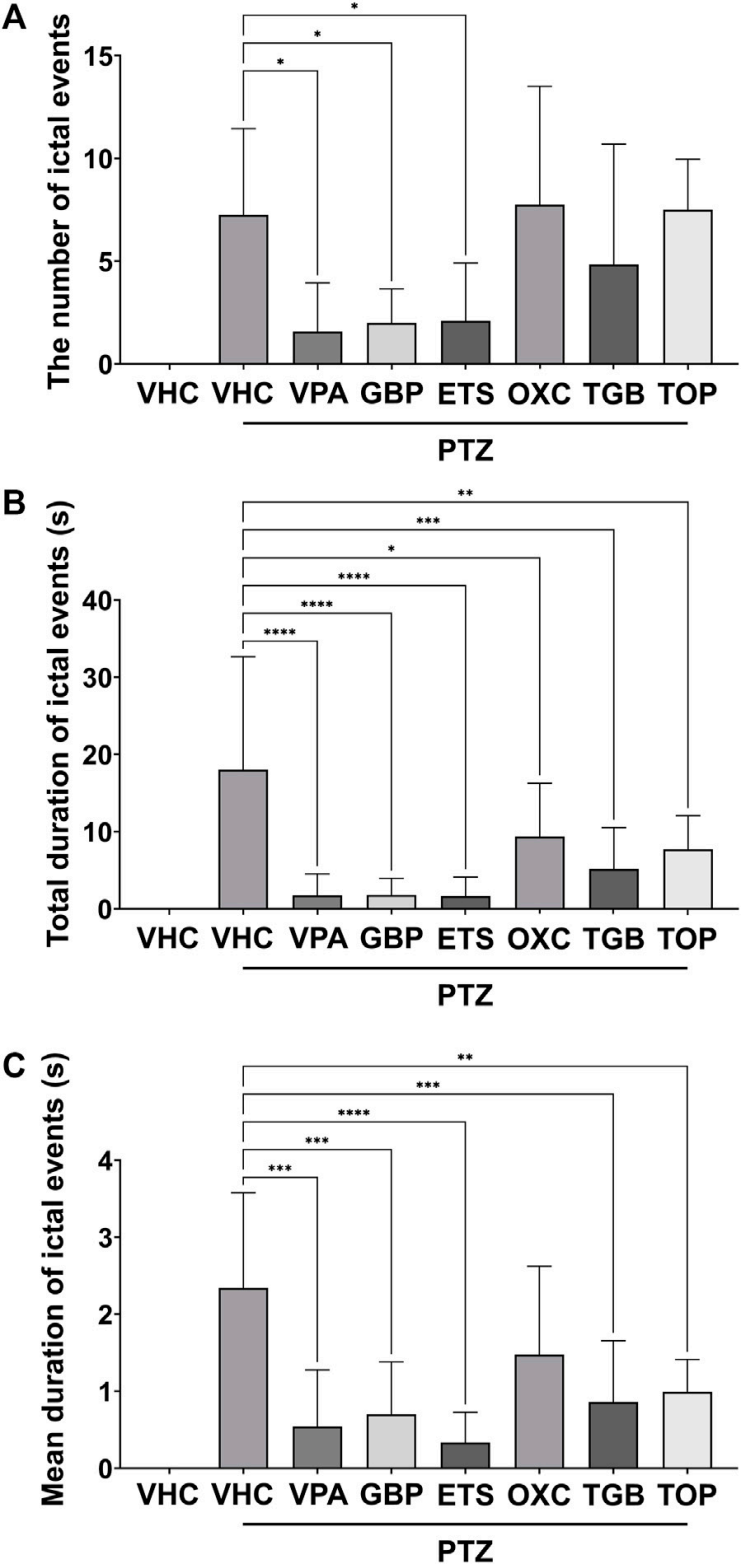
Quantitative analysis of ictal events based on one-way ANOVA of the EEG data recorded for 20 min from eight groups: the vehicle (VHC) and PTZ (VHC + PTZ) groups as well as and six groups cotreated with AEDs and PTZ (*n* = 8 per group). All AEDs were administered with a high dose (VPA: 124 mpk, GBP: 168 mpk, ETS: 108 mpk, OXC: 45.6 mpk, TGB: 2.6 mpk, TOP: 42.9 mpk). **(A)** Number of ictal events in each group. **(B)** Total duration of ictal events in each group. **(C)** Mean duration of ictal events in each group. Significant differences are denoted as follows: *p < 0.05, **p < 0.01, ***p < 0.001, and ****p < 0.0001. Raw statistical results are summarized in Supplementary Table S3.

As shown in Figure 2A, the VPA, GBP, and ETS groups showed a statistically significant decrease in the number of ictal events compared to the PTZ-only group. In the OXC, TGB and TOP groups, the number of ictal events was similar to or less than that of the PTZ-only group, but these differences were not statistically significant. As shown in Figure 2B, the total duration of ictal events decreased significantly in all six groups (the VPA, GBP, ETS, OXC, TGB, and TOP groups). Figure 2C confirms that the mean duration of ictal events in the VPA, GBP, ETS, TGB, and TOP groups decreased significantly; the mean duration in the OXC group was decreased compared to that in the PTZ-only group, but the difference was not statistically significant. Based on these results, all AEDs were confirmed to induce a statistically significant difference in at least one out of the three epilepsy indicators compared to those in the PTZ-only group.

**FIGURE 3:**
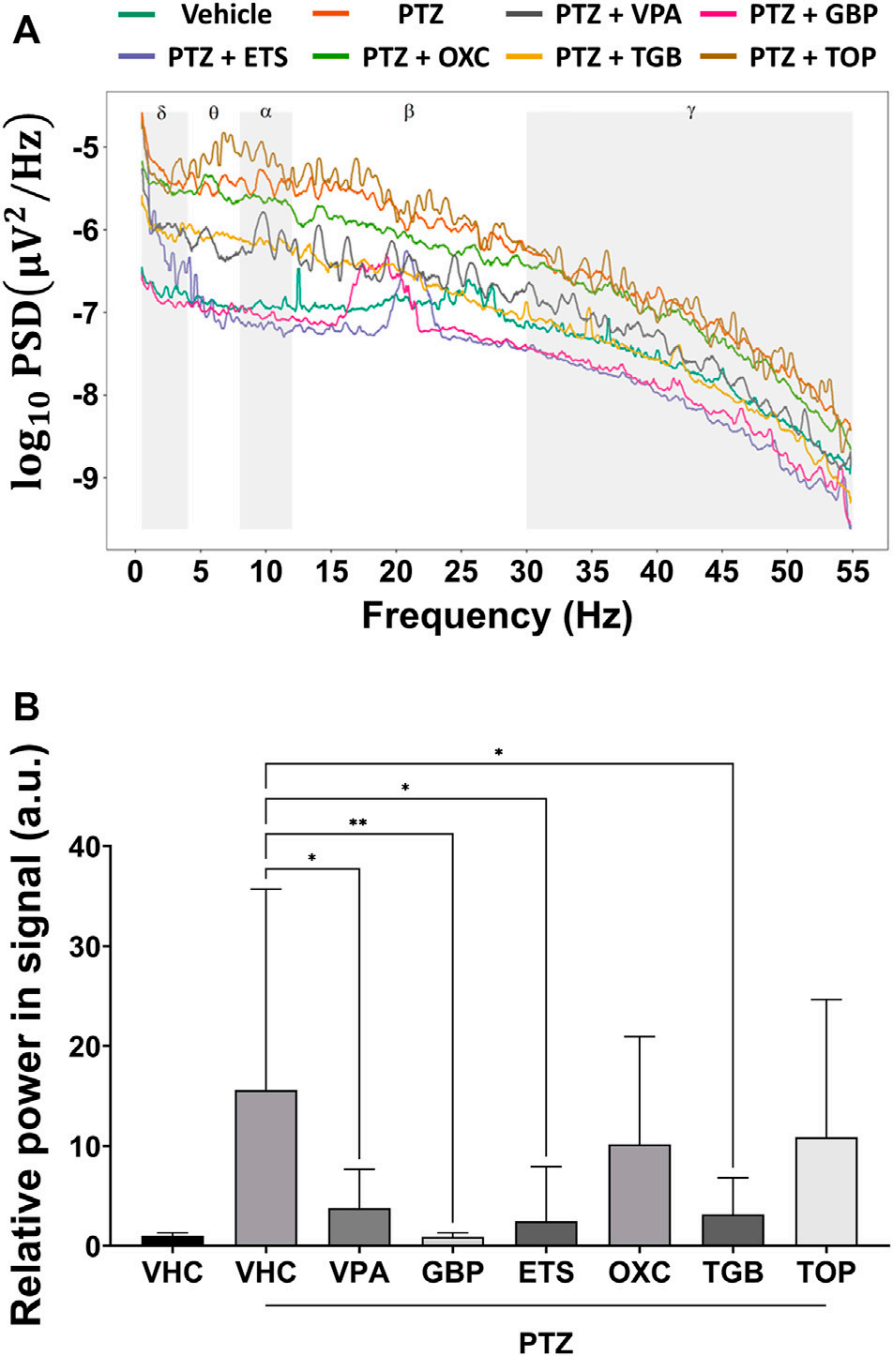
Analysis of the power spectral density based on one-way ANOVA of the EEG signals obtained from each group (*n* = 8 per group). **(A)** The average power spectral density of each group is represented on a log scale over the frequency range from 0.5 to 55 Hz. **(B)** The relative power is obtained by normalizing the power contained in the EEG signal from each group by the power contained in the EEG signal from the control group (VHC). Significant differences are denoted as follows: *p < 0.05, **p < 0.01, ***p < 0.001, and ****p < 0.0001. Raw statistical results are summarized in Supplementary Table S4.

As shown in Figure 3A, the PSD of the PTZ-only group was higher than that of the control group over all frequency bands from 0.5 to 55 Hz. The PSD of the VPA, GBP, EST and TGB groups decreased compared to that of the PTZ-only group, but that of the OXC and TOP groups did not. In Figure 3B, the total power contained in the signal of each group was obtained by integrating the PSD over all measured frequencies, and the relative power was obtained by normalizing the total power of each group according to the total power of the control group. As shown in Figure 3B, the VPA, GBP, ETS, and TGB groups exhibited significantly decreased relative power compared to the PTZ-only group, whereas the OXC and TOP groups did not.

AEDs were judged to be effective if they showed a statistically significant difference in at least one of three indicators of the number, total duration and mean duration of ictal events. According to a previous study of AEDs that used zebrafish (Afrikanova et al., 2013), as summarized in Table 1, evaluating the efficacy of GBP and TOP through behavioral analysis may produce false negatives, which we could detect through EEG analysis. Previous behavior-based screening of AEDs reports false positive rates of up to 80%, and EEG-based screening significantly reduces false positives and false negatives (Baraban et al., 2013; Eimon et al., 2018). Our assay based on EEG from adult zebrafish could significantly reduce false negatives. Using the efficacy assay method proposed in the current study, the AED efficacy could be observed in all six drugs including the three drugs that had not shown the efficacy in previous studies. One explanation why the findings in the present study differ from those of the previous zebrafish EEG study is the age of the experimental animals used. The previous study used larvae (Afrikanova et al., 2013), whereas we used adults. On the other hand, the zebrafish EEG data obtained in the present study seemed to be highly correlated with the previous rodent EEG data (Watanabe et al., 2010; Afrikanova et al., 2013; Yuen and Trocóniz, 2015).

**TABLE 1:** Comparison of the efficacy of AEDs using PTZ-induced zebrafish and rodent models. The symbols “+” and “−” indicate that the AED was or was not effective, respectively. NA indicates a lack of data on the efficacy of a given AED (Nielsen et al., 1991; Baumeister et al., 2008; Hitt et al., 2010; Afrikanova et al., 2013).

	Zebrafish model			Rodent model
	EEG (this study)	Behavior	EEG	EEG
VPA	+	+	+	+
GBP	+	−	+	+
ETS	+	+	−	+
OXC	+	+	−	NA
TGB	+	+	−	NA
TOP	+	−	+	−

The sensitivities of AED analyses in zebrafish using behavioral and EEG methods were 66% and 50%, respectively, based on previous studies. In our study, anti-seizure efficacy was confirmed in all AEDs tested, indicating a sensitivity of 100%. We quantitatively analyzed all three indicators of ictal events: the number, total duration, and mean duration. If at least one out of three indicators showed statistically significant differences, the drug was deemed effective. This suggests that measuring all three indicators of ictal events is a good way of excluding false negatives when studying the effectiveness of AEDs.

### Ictal events in the EEG are quantitative biomarkers of AED efficacy

**FIGURE 4:**
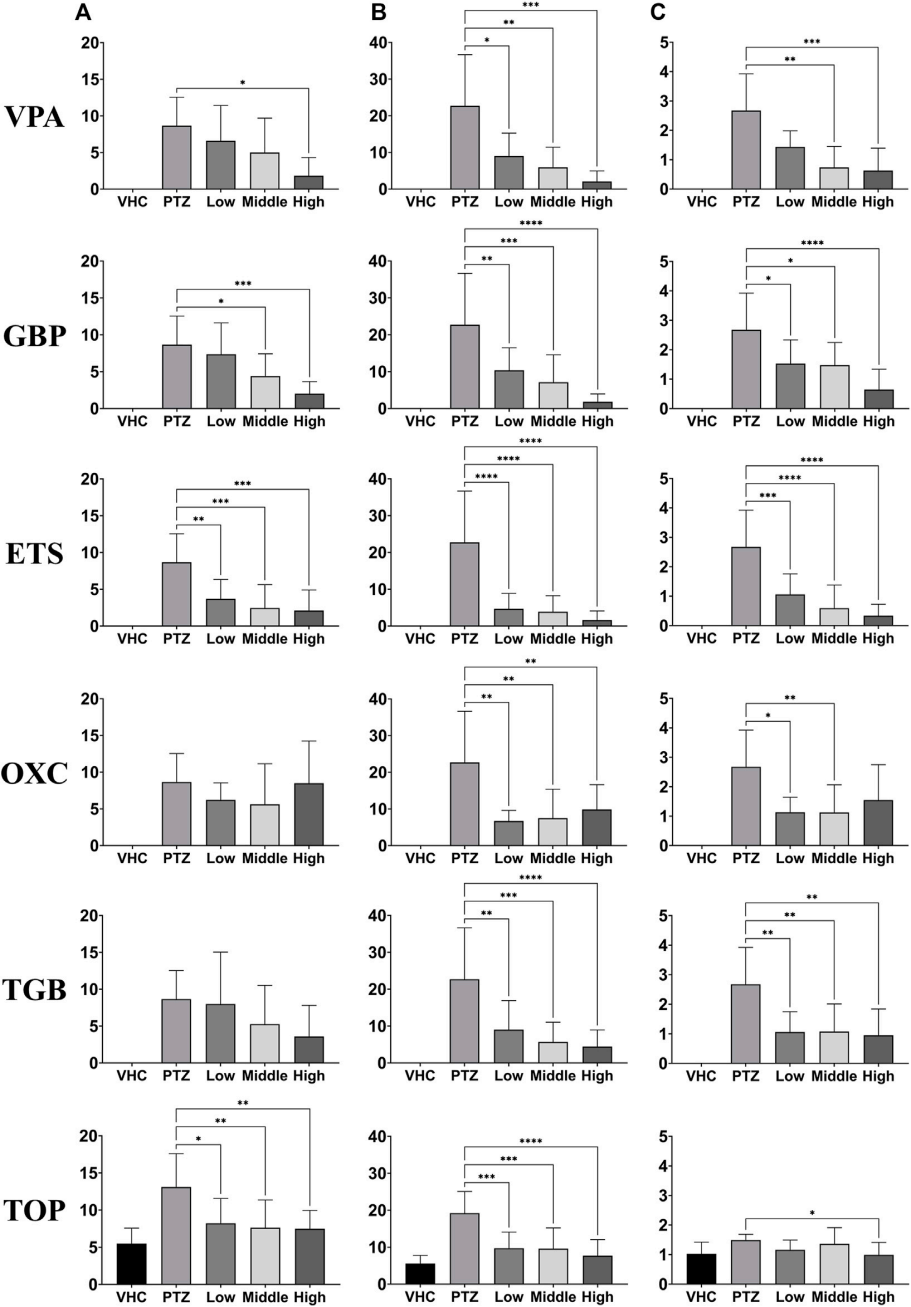
Evaluation of the responses depending on dose of six AEDs (VPA, GBP, ETS, OXC, TGB, and TOP, n = 8 per group) based on one-way ANOVA. EEG data were recorded for 20 min in each group at each dose. The three dosages used for each AED are listed in Supplementary Table S1. **(A)** Number of ictal-like events. **(B)** Total duration of ictal-like events. **(C)** Mean duration of ictal-like events. Significant differences are denoted as follows: *p < 0.05, **p < 0.01, ***p < 0.001, and ****p < 0.0001. Raw statistical results are summarized in Supplementary Table S5.

We recorded EEG signals from the zebrafish model of PTZ-induced seizures after administering different doses of six AEDs. Three doses were chosen for each drug based on the doses used in previous studies on mice (Nielsen et al., 1991; Czuczwar et al., 1999; Hwang et al., 2020). From the recorded EEG data for each group, the number, total duration, and mean duration of ictal events were determined, as shown in Figure 4. Each group consisted of 40 animals, with 8 animals for each of the five conditions (control, PTZ-only, and low, medium and high doses of each AED). For all AEDs, statistically significant reductions were observed in at least one of the three indicators of ictal events. The VPA, GBP, and ETS groups showed a clear pattern of decrease in all three indicators as the dose increased. The OXC group showed a statistically significant decrease in the total duration and mean duration of ictal events compared to the PTZ-only group but did not show a trend depending on dose. The TGB group exhibited a statistically significant decrease in the total duration and mean duration, with an effect depending on dose confirmed in the total duration, but the number of ictal events did not decrease significantly. The TOP group showed a statistically significant decrease in the number and total duration of ictal events compared to the PTZ-only group, but with no clear effect of dose.

The use of adult zebrafish has several advantages, including the possibility of oral drug administration, which enables accurate drug delivery to experimental animals, resulting in more precise PTZ models and more accurate drug evaluations (Shank et al., 1994; Hwang et al., 2020). In preclinical tests using rodents, drugs are delivered through oral or intraperitoneal administration. On the other hand, the way to deliver drugs to larvae is limited: the whole body of larvae is exposed in a medium containing drugs. Such differences in delivering drugs to different animal models have made quantitative comparison and prediction difficult among different species. However, our study demonstrated that AED screening using adult zebrafish can be quantitatively compared with the results obtained from rodents. The current study also recorded EEG data using a non-invasive method that reduced stress compared to the invasive EEG recordings used in the previous study (Afrikanova et al., 2013). In their study, behavioral observation was conducted for 30 min, and EEG data were separately recorded for 10 min; in contrast, we recorded EEG data for 20 min. Therefore, the current study was able to obtain more EEG data by extending the length of EEG recording.

By using EEG signals, the efficacy of various AEDs depending on dose was confirmed. Based on our results, ictal brain signals were confirmed as a quantitative indicator of the antiseizure efficacy of AEDs.

### The effective dose can be determined based on EEG data

**FIGURE 5:**
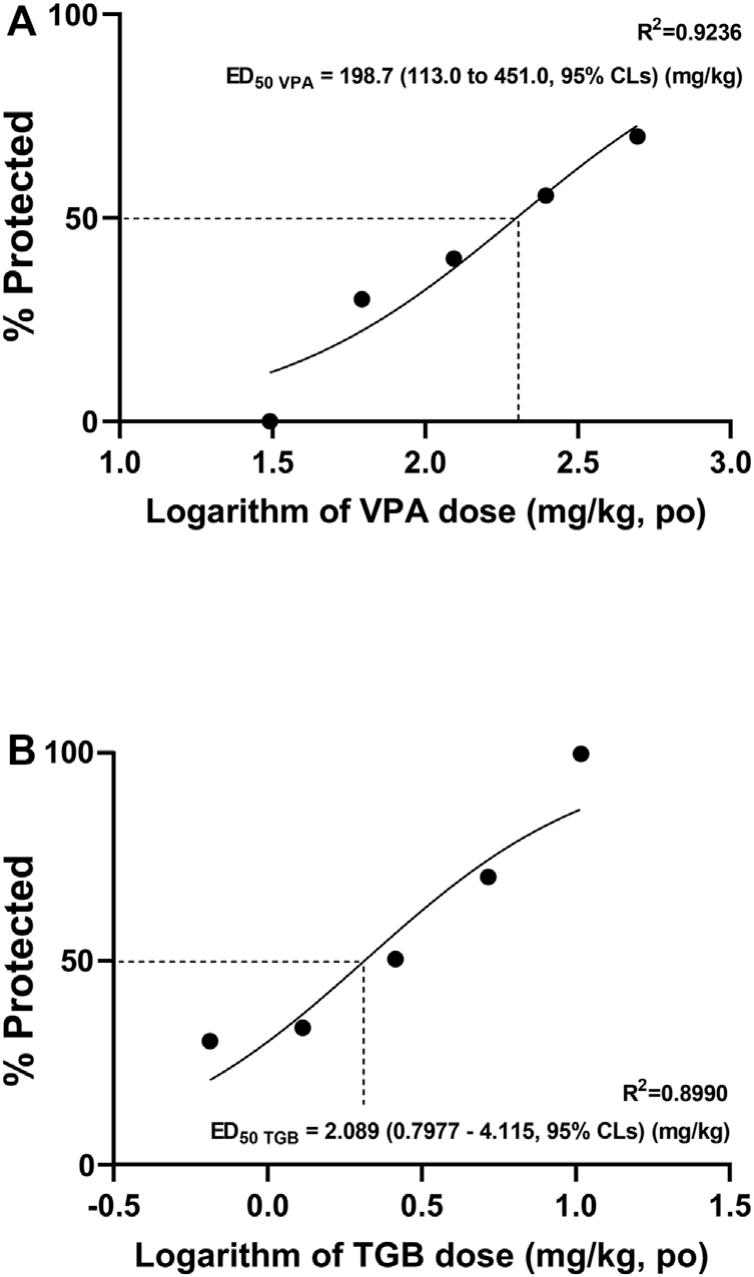
Evaluation of the efficacy of VPA and TGB based on ED50 data. The ED50 curves were obtained using 5 doses based on the three indices of ictal events (number, total duration, and mean duration) for **(A)** VPA and **(B)** TGB.

We selected VPA and TGB, the drugs that showed a clear effect on all three indicators of ictal events depending on dose, to obtain ED50 values. Additionally, VPA and TGB represented drugs with a high ED50 value and a low ED50 value, respectively. To obtain the ED50 value for each drug, EEG signals were recorded at 5 different doses. Forty zebrafish were used per drug, with 8 fish receiving each of the 5 doses. The ED50 values in Figure 5 were plotted according to the proportion of positive samples in the group. We compared the average values of the three indicators of ictal event from the PTZ-only group with the values of the three ictal event indicators from a given sample: if at least one of these values was at 50% of the PTZ-only value or less, the sample was labeled positive for AED efficacy. The ED50 values of VPA and TGB were determined to be 198.7 mg/kg and 2.089 mg/kg, respectively.

According to a previous study using behavioral analysis (Nieoczym et al., 2012), the ED50 values for VPA and TGB in mice were 121.4 mg/kg and 0.88 mg/kg, respectively. The difference in the ED50 values between the present study and the previous study is likely due to the differences in the species and the models used. The PTZ model used in this study was induced by oral administration, but the PTZ model used in the mouse study was induced through intraperitoneal administration. Despite such differences, the ED50 values obtained from zebrafish (*via* EEG) and mice (*via* behavioral analysis) showed a consistent tendency.

We obtained ED50 curves from ictal EEG signals and confirmed that AED effectiveness could be quantitatively compared and evaluated. Additionally, we proposed a method for calculating ED50 values using ictal signals. We obtained the ED50 values of VPA and TGB from the ED50 curves, which resulted in ED50 data similar to those reported in the previous study (Nieoczym et al., 2012) that tested drugs with high and low ED50 values. The ratio of the ED50 values of VPA and TGB was similar to that obtained in the previous study, although the ED50 values of VPA and TGB that we obtained from zebrafish using EEG signals were 1.60 times and 2.37 times higher than those obtained from mice, respectively. The higher ED50 values may be due to the dose of PTZ, which was higher in our study; thus, the PTZ-induced model used in the present study was a more severe epilepsy model than that used in the previous study.

### Significance and limitations of the study

It is noteworthy that the present study is the first to report ED50 values from zebrafish EEG analysis. Previously, ED50 values in zebrafish were reported only from behavioral analysis. We could obtain such quantitative results because we used adult zebrafish. Previously only larval zebrafish were used in drug screening (Afrikanova et al., 2013; Hong et al., 2016; Tomasello and Sive, 2020). Our study is the first to screen AEDs using adult zebrafish, which have the fully developed nervous system and therefore can model neurological diseases in mature organisms. Delivering drugs to adult zebrafish through oral administration enables quantitative inter-species comparison of drug efficacy, whereas the whole body of larvae needs to be exposed to drugs, which makes it difficult to compare the data obtained from larvae with the data obtained from rodents where oral or intraperitoneal administration is used.

Previous EEG studies used invasive electrodes to penetrate the skin of animals (Pineda et al., 2011; Hong et al., 2016) while the present study employs non-invasive EEG measurement. In more recent studies, non-invasive EEG measurement from adult zebrafish has been reported: for instance, the concept of simultaneous EEG measurement from multiple animals was reported (Lee et al., 2020), and the effect of a new anti-seizure drug was evaluated using non-invasive EEG (Hwang et al., 2020). However, there has been no systematic comparative study on the efficacy of various AEDs using non-invasive EEG signals from adult zebrafish, which was first demonstrated in the current study.

However, there are limitations of the study. Since the disease model we used was an acute-induced seizure model, the feasibility of evaluating the drug efficacy using EEG for other epilepsy models needs to be investigated in future studies. In addition, we used only males, as it is known to be difficult to obtain consistent results using females due to the strong influence of hormones. Thus, the evaluation of the efficacy of AEDs using the proposed method was not verified for females. Further studies would need to include both males and females.

## Conclusion

We successfully demonstrated that the efficacy of AEDs can be quantitatively assessed through EEG from adult zebrafish, providing quantitative responses depending on dose and ED50 data. The tendency in seizure suppression depending on dose was clearly demonstrated with four out of six AEDs tested. Also, we could obtain the ED50 data for two representative AEDs, which provides the effective dose for each drug. Additionally, the proposed EEG assay can detect false-negatives that cannot be detected when conventional behavioral analysis is used. Considering that EEG is the clinical standard in diagnosing epilepsy, our study proposes a new way to evaluate the efficacy of AEDs in a way that is closer to the clinically used standard method even at the early stage of drug development. The zebrafish EEG assay proposed in this study enables the efficacy evaluation more similar to those used for preclinical AED assays using mammals than previous zebrafish studies, and is thus expected to reduce animal experiments using mammals. As the result, assessments of AED efficacy using zebrafish EEG are expected to facilitate the development of drugs to treat epilepsy and provide statistical and quantitative data for large-scale drug screening.

## Data Availability

The original contributions presented in the study are included in the article/Supplementary Material, further inquiries can be directed to the corresponding author.
