# Supplementary material for: Zebrafish EEG predicts the efficacy of antiepileptic drugs
Source: Front Pharmacol. 2022 Dec 8;13:1055424. doi: 10.3389/fphar.2022.1055424 (PMC9772616; doi:10.3389/fphar.2022.1055424)
Supplement: Supplementary file 1 [file DataSheet1.pdf]

Supplementary Material

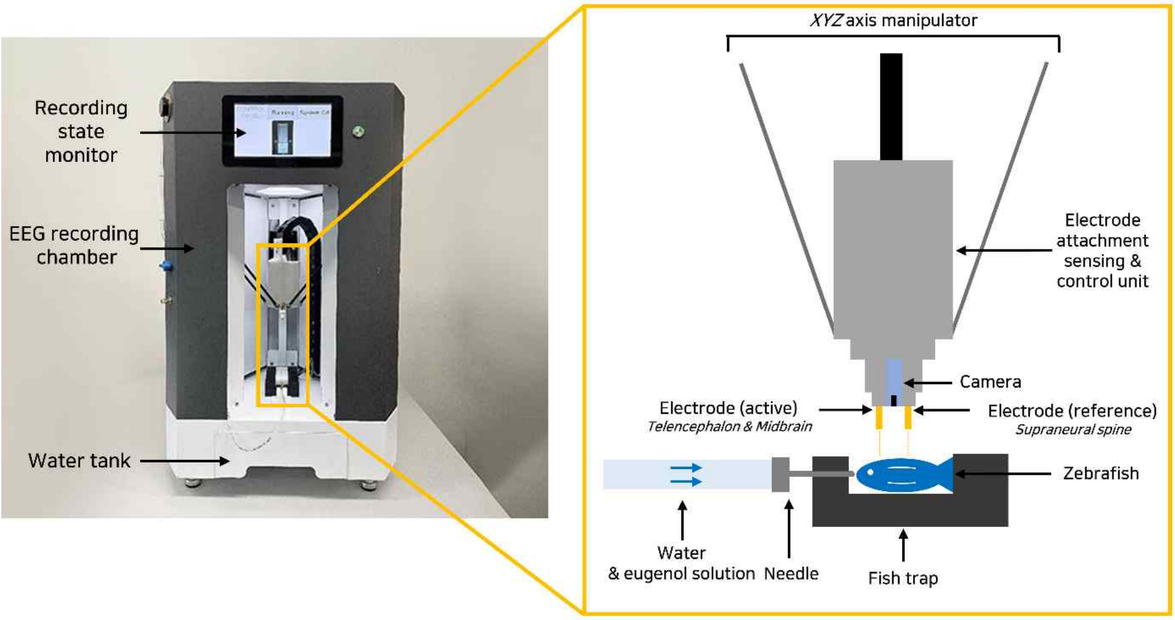

Figure S1. Zebrafish EEG measurement system. Electrodes were attached to the heads of zebrafish that were immobilized in the fish trap as the location of the head was determined through an internal camera. The inside of the system was sealed with a shielding film to reduce electromagnetic interference and noise. During recording of EEG data, the system was turned off to record the data with minimal electromagnetic interference.

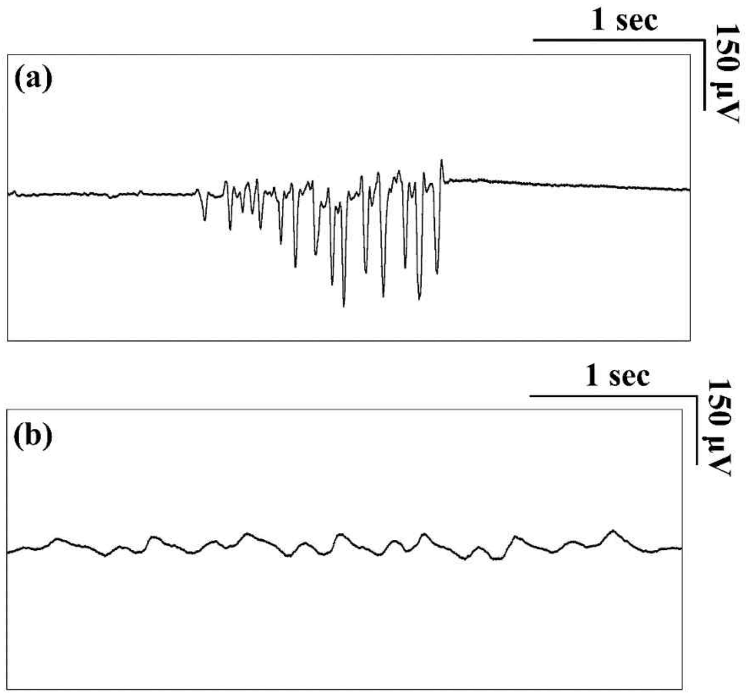

Figure S2. Examples of measured zebrafish EEG signals representing (a) tonic-clonic seizure and (b) absence-like seizure.

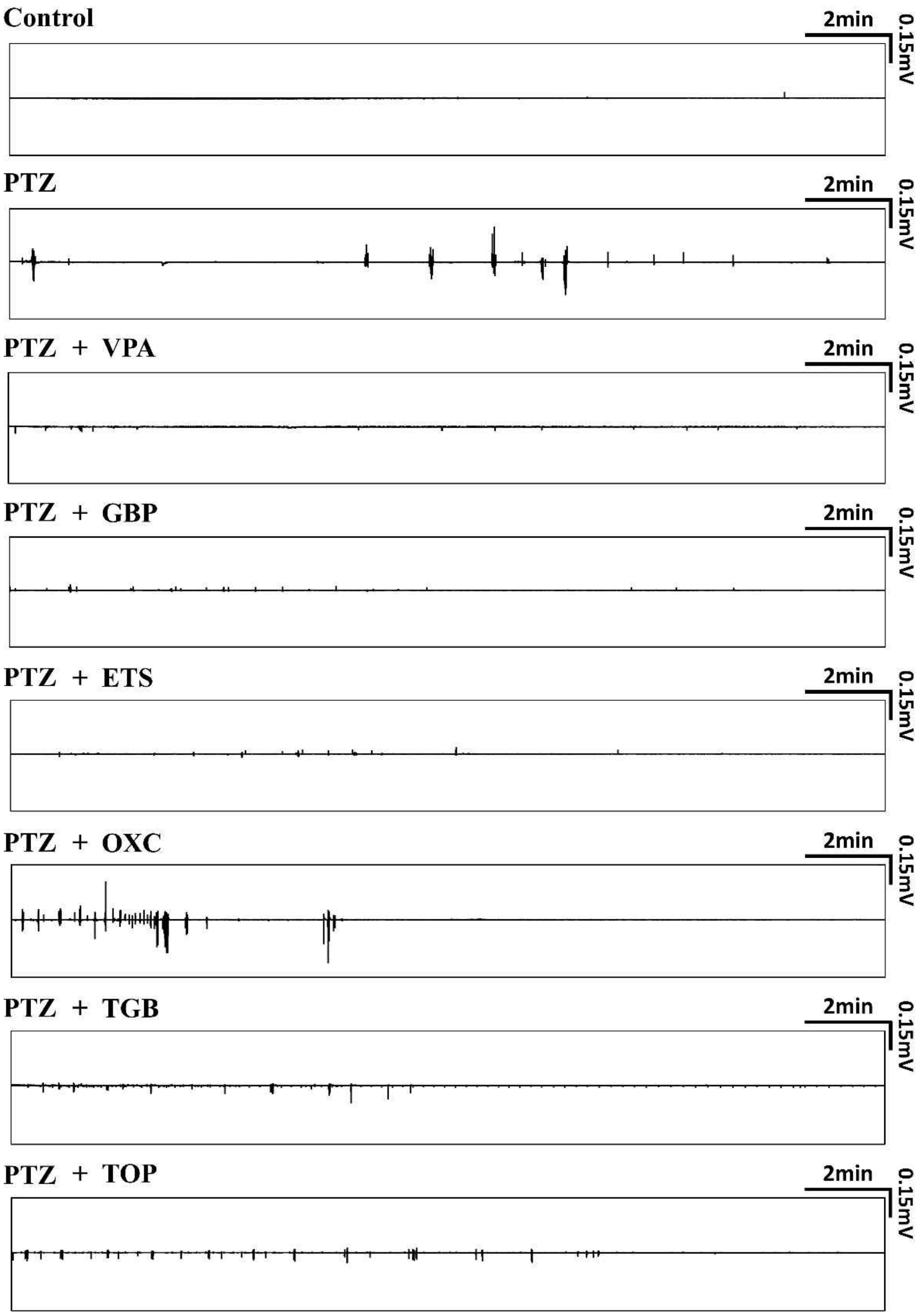

Figure S3. Representative real-time EEG recordings for 20 minutes from all groups.

Table S1. Dosage of each AED, taken from the literature (Yien and Trocóniz, 2015; Czuczwar et al., 1999; Nielsen et al., 1991). The efficacy assay was performed with the high dose of AEDs from ED50 values reported in previous studies. In addition, when the efficacy was not observed in experiments, the dose of AEDs was increased to twice the high dose. Low and middle doses were defined to be 1/4 and 1/2 of the high dose, respectively.

	**Low (mpk)**	**Middle (mpk)**	**High (mpk)**
**VPA** (Czuczwar et al., 1999)	**31**	**62**	**124**
**GBP** (Yien and Trocóniz, 2015)	**42**	**84**	**168**
**ETS** (Czuczwar et al., 1999)	**27**	**54**	**108**
**OXC** (Yien and Trocóniz, 2015)	**11.4**	**22.8**	**45.6**
**TGB** (Nielsen et al., 1991)	**0.65**	**1.3**	**2.6**
**TOP** (Yien and Trocóniz, 2015)	**10.725**	**21.45**	**42.9**

Table S2. Characteristics of ictal events in zebrafish, rodents, and humans (Cho et al., 2019; Amzica and Steriade, 1998).

	**Zebrafish**		**Rodents**		**Humans**	
**Seizure type**	**Tonic-clonic**	**Absence**	**Tonic-clonic**	**Absence**	**Tonic-clonic**	**Absence**
**Amplitude**	**High**	**Low**	**High**	**Low**	**High**	**Low**
**Frequency (Hz)**	**5~7**	**2~3**	**6~8**	**3~7**	**4~5**	**~3**

Hereafter, Tables S3 to S5 summarize all statistical results of one-way analysis of variance (ANOVA) and normality test of all datasets presented in Figure 2 to 4. The method of one-way ANOVA and the interpretation of the results are explained in the main text. Normality tests (Kolmogorov-Smirnov test and Shapiro-Wilk test) were performed to check the normality in all datasets. The Kolmogorov-Smirnov test is a goodness-of-fit method that compares the maximum distance between an experimental cumulative distribution function and a theoretical cumulative distribution function. The Kolmogorov–Smirnov statistics quantify the distance between the empirical distribution function of a sample and the cumulative distribution function of a reference distribution, or between the empirical distribution function of two samples, which have values between 0 and 1. If the p-value provided by the Kolmogorov-Smirnov test is $p < 0.05$, then the test rejects the null hypothesis that the variable is normally distributed. The Shapiro-Wilk test can be used to determine whether a sample fits a normal distribution and is usually used for small samples. The Shapiro-Wilk statistic values are basically a measure of how well an ordered and standardized sample quantile fits the standard normal quantiles, which range from 0 and 1, with 1 being a perfect match. The test rejects the normality hypothesis when the p-value is less than or equal to 0.05.

Table S3. Raw statistical results of the datasets presented in Figure 2. All data were analyzed by one-way ANOVA (F value), followed by Dunnett's test (post hoc). All adjusted p-values were the result of Dunnett's test. Statistical significance was accepted for $p < 0.05$, with indications of * for $p<0.05$, ** for $p<0.01$, and *** for $p<0.001$. Normality tests (Kolmogorov-Smirnov test and Shapiro-Wilk test) were performed to check the normality in all datasets. Statistics of the Kolmogorov-Smirnov test have values between 0 and 1, with 1 being the maximum. Statistics of the Shapiro-Wilk test have values between 0 and 1, with 1 being a perfect match. 'Sig.' represents the p-value in normality tests.

<table>
<tr><td rowspan="16">Figure 2A</td><td colspan="5">One-way ANOVA test</td></tr>
<tr><td>Dunnett's multiple comparisons test</td><td>Summary</td><td>Adjusted P Value</td><td rowspan="8">ANOVA summary</td><td>F-value</td></tr>
<tr><td>PTZ vs. VHC</td><td>**</td><td>0.0021</td><td rowspan="7">6.061</td></tr>
<tr><td>PTZ vs. VPA</td><td>*</td><td>0.0309</td></tr>
<tr><td>PTZ vs. GBP</td><td>*</td><td>0.0214</td></tr>
<tr><td>PTZ vs. ETS</td><td>*</td><td>0.0334</td></tr>
<tr><td>PTZ vs. OXC</td><td>ns</td><td>0.9996</td></tr>
<tr><td>PTZ vs. TGB</td><td>ns</td><td>0.5747</td></tr>
<tr><td>PTZ vs. TOP</td><td>ns</td><td>0.9998</td></tr>
<tr><td colspan="5">Tests of normality</td></tr>
<tr><td rowspan="2">Group</td><td colspan="2">Kolmogorov-Smirnov test</td><td colspan="2">Shapiro-Wilk test</td></tr>
<tr><td>Statistic</td><td>Sig.</td><td>Statistic</td><td>Sig.</td></tr>
<tr><td>VHC</td><td>ns</td><td>ns</td><td>1</td><td><0.0001</td></tr>
<tr><td>PTZ</td><td>0.9008</td><td>0.2937</td><td>0.2039</td><td>>0.1000</td></tr>
<tr><td>VPA</td><td>0.6754</td><td>0.002</td><td>0.3177</td><td>0.0314</td></tr>
<tr><td>GBP</td><td>0.927</td><td>0.3499</td><td>0.1443</td><td>>0.1000</td></tr>
</table>

	ETS	0.7935	0.0121	0.2728	0.0335
	OXC	0.9579	0.7531	0.1493	>0.1000
	TGB	0.808	0.0116	0.2061	>0.1000
	TOP	0.9231	0.3834	0.1805	>0.1000
**Figure 2B**	**One-way ANOVA test**				
	Dunnett's multiple comparisons test	Summary	Adjusted P Value	ANOVA summary	F-value
	PTZ vs. VHC	****	<0.0001		8.144
	PTZ vs. VPA	****	<0.0001		
	PTZ vs. GBP	****	<0.0001		
	PTZ vs. ETS	****	<0.0001		
	PTZ vs. OXC	*	0.0155		
	PTZ vs. TGB	***	0.0001		
	PTZ vs. TOP	**	0.004		
	**Tests of normality**				
	Group	Kolmogorov-Smirnov test		Shapiro-Wilk test	
		Statistic	Sig.	Statistic	Sig.
	VHC	ns	ns	1	<0.0001
	PTZ	0.899	0.2831	0.1998	>0.1000
	VPA	0.7386	0.0096	0.3103	0.0404
	GBP	0.8073	0.0114	0.2041	>0.1000
	ETS	0.7418	0.0028	0.3058	0.0086
	OXC	0.9371	0.461	0.1654	>0.1000
	TGB	0.8616	0.0511	0.2571	0.0276
	TOP	0.9293	0.4413	0.1867	>0.1000
**Figure 2C**	**One-way ANOVA test**				
	Dunnett's multiple comparisons test	Summary	Adjusted P Value	ANOVA summary	F-value
	PTZ vs. VHC	****	<0.0001		7.391
	PTZ vs. VPA	***	0.0002		
	PTZ vs. GBP	***	0.0001		
	PTZ vs. ETS	****	<0.0001		
	PTZ vs. OXC	ns	0.0871		
	PTZ vs. TGB	***	0.0006		
	PTZ vs. TOP	**	0.0032		
	**Tests of normality**				
	Group	Kolmogorov-Smirnov test		Shapiro-Wilk test	
		Statistic	Sig.	Statistic	Sig.
	VHC	ns	ns	1	<0.0001

	PTZ	0.9827	0.975	0.1204	>0.1000
	VPA	0.7525	0.0135	0.3402	0.0141
	GBP	0.8553	0.0427	0.2507	0.0359
	ETS	0.7808	0.0085	0.3045	0.0092
	OXC	0.7708	0.0044	0.278	0.011
	TGB	0.8768	0.0798	0.1781	>0.1000
	TOP	0.9321	0.469	0.2092	>0.1000

Table S4. Raw statistical results of the datasets presented in Figure 3. All data were analyzed by one-way ANOVA (F value), followed by Dunnett's test (post hoc). All adjusted p-values were the result of Dunnett's test. Statistical significance was accepted for $p < 0.05$, with indications of * for $p<0.05$, ** for $p<0.01$, and *** for $p<0.001$. Normality tests (Kolmogorov-Smirnov test and Shapiro-Wilk test) were performed to check the normality in all datasets. Statistics of the Kolmogorov-Smirnov test have values between 0 and 1, with 1 being the maximum. Statistics of the Shapiro-Wilk test have values between 0 and 1, with 1 being a perfect match. 'Sig.' represents the p-value in normality tests.

**Figure 3B**	**One-way ANOVA test**				
	Dunnett's multiple comparisons test	Summary	Adjusted P Value		F-value
	PTZ vs. VHC	**	0.0059	ANOVA summary	3.507
	PTZ vs. VPA	*	0.0273		
	PTZ vs. GBP	**	0.0036		
	PTZ vs. ETS	*	0.0161		
	PTZ vs. OXC	ns	0.6003		
	PTZ vs. TGB	*	0.018		
	PTZ vs. TOP	ns	0.7769		
	**Tests of normality**				
	Group	Kolmogorov-Smirnov test		Shapiro-Wilk test	
		Statistic	Sig.	Statistic	Sig.
	VHC	0.8856	0.2127	0.2058	>0.1000
	PTZ	0.8083	0.0351	0.2637	>0.1000
	VPA	0.7333	0.0085	0.3408	0.0137
	GBP	0.9522	0.6688	0.1323	>0.1000
	ETS	0.3953	<0.0001	0.5001	<0.0001
	OXC	0.8261	0.0189	0.2818	0.0092
	TGB	0.7171	0.0012	0.2968	0.0045
	TOP	0.6992	0.0014	0.3432	0.003

Table S5. Raw statistical results of the datasets presented in Figure 4. All data were analyzed by one-way ANOVA (F value), followed by Dunnett's test (post hoc). All adjusted p-values were the result of Dunnett's test. Statistical significance was accepted for $p < 0.05$, with indications of * for $p<0.05$, ** for $p<0.01$, and *** for $p<0.001$. Normality tests (Kolmogorov-Smirnov test and Shapiro-Wilk test) were performed to check the normality in all datasets. Statistics of the Kolmogorov-Smirnov test have values between 0 and 1, with 1 being the maximum. Statistics of the Shapiro-Wilk test have values between 0 and 1, with 1 being a perfect match. 'Sig.' represents the p-value in normality tests.

**Figure 4A VPA**	**One-way ANOVA test**				
	Dunnett's multiple comparisons test	Summary	Adjusted P Value	ANOVA summary	F-value
	PTZ vs. VHC	**	0.0025		5.194
	PTZ vs. Low (VPA)	ns	0.7601		
	PTZ vs. Middle (VPA)	ns	0.3061		
	PTZ vs. High (VPA)	*	0.0122		
	**Tests of Normality**				
	Group	Kolmogorov-Smirnov test		Shapiro-Wilk test	
		Statistic	Sig.	Statistic	Sig.
	VHC	ns	ns	1	<0.0001
	PTZ	0.9008	0.2937	0.2039	>0.1000
	Low (VPA)	0.8232	0.1236	0.3049	>0.1000
	Middle (VPA)	0.836	0.1542	0.2651	>0.1000
	High (VPA)	0.6754	0.002	0.3177	0.0314
**Figure 4B VPA**	**One-way ANOVA test**				
	Dunnett's multiple comparisons test	Summary	Adjusted P Value	ANOVA summary	F-value
	PTZ vs. VHC	***	0.0003		7.895
	PTZ vs. Low (VPA)	*	0.0256		
	PTZ vs. Middle (VPA)	**	0.0056		
	PTZ vs. High (VPA)	***	0.0005		
	**Tests of Normality**				
	Group	Kolmogorov-Smirnov test		Shapiro-Wilk test	
		Statistic	Sig.	Statistic	Sig.
	VHC	ns	ns	1	<0.0001
	PTZ	0.899	0.2831	0.1998	>0.1000
	Low (VPA)	0.933	0.6171	0.2194	>0.1000
	Middle (VPA)	0.7742	0.0491	0.2976	>0.1000
	High (VPA)	0.7386	0.0096	0.3103	0.0404
	**One-way ANOVA test**				

Figure 4C VPA

Dunnett's multiple comparisons test	Summary	Adjusted P Value		F-value
PTZ vs. VHC	****	<0.0001	ANOVA summary	9.252
PTZ vs. Low (VPA)	ns	0.0578		
PTZ vs. Middle (VPA)	**	0.0021		
PTZ vs. High (VPA)	***	0.0008		

Tests of Normality

Group	Kolmogorov-Smirnov test		Shapiro-Wilk test	
	Statistic	Sig.	Statistic	Sig.
VHC	ns	ns	1	<0.0001
PTZ	0.9827	0.975	0.1204	>0.1000
Low (VPA)	0.7932	0.0713	0.3766	0.0194
Middle (VPA)	0.8711	0.271	0.2492	>0.1000
High (VPA)	0.7525	0.0135	0.3402	0.0141

Figure 4A GBP

One-way ANOVA test

Dunnett's multiple comparisons test	Summary	Adjusted P Value		F-value
PTZ vs. VHC	***	0.0001	ANOVA summary	9.809
PTZ vs. Low (GBP)	ns	0.7905		
PTZ vs. Middle (GBP)	*	0.0334		
PTZ vs. High (GBP)	***	0.0004		

Tests of Normality

Group	Kolmogorov-Smirnov test		Shapiro-Wilk test	
	Statistic	Sig.	Statistic	Sig.
VHC	ns	ns	1	<0.0001
PTZ	0.9008	0.2937	0.2039	>0.1000
Low (GBP)	0.7228	0.0009	0.2614	0.0344
Middle (GBP)	0.872	0.1056	0.2526	0.0701
High (GBP)	0.927	0.3499	0.1443	>0.1000

Figure 4B GBP

One-way ANOVA test

Dunnett's multiple comparisons test	Summary	Adjusted P Value		F-value
PTZ vs. VHC	****	<0.0001	ANOVA summary	11.03
PTZ vs. Low (GBP)	**	0.0042		
PTZ vs. Middle (GBP)	***	0.0004		
PTZ vs. High (GBP)	****	<0.0001		

Tests of Normality

Group	Kolmogorov-Smirnov test	Shapiro-Wilk test

		Statistic	Sig.	Statistic	Sig.
	VHC	ns	ns	1	<0.0001
	PTZ	0.899	0.2831	0.1998	>0.1000
	Low (GBP)	0.8933	0.1528	0.1671	>0.1000
	Middle (GBP)	0.6876	0.0006	0.3652	0.0005
	High (GBP)	0.8073	0.0114	0.2041	>0.1000
**Figure 4C GBP**	**One-way ANOVA test**				
	Dunnett's multiple comparisons test	Summary	Adjusted P Value	ANOVA summary	F-value
	PTZ vs. VHC	****	<0.0001		10.07
	PTZ vs. Low (GBP)	*	0.0231		
	PTZ vs. Middle (GBP)	*	0.0193		
	PTZ vs. High (GBP)	****	<0.0001		
	**Tests of Normality**				
	Group	Kolmogorov-Smirnov test		Shapiro-Wilk test	
		Statistic	Sig.	Statistic	Sig.
	VHC	ns	ns	1	<0.0001
	PTZ	0.9827	0.975	0.1204	>0.1000
	Low (GBP)	0.8936	0.1541	0.2368	0.0852
	Middle (GBP)	0.9192	0.3506	0.1781	>0.1000
	High (GBP)	0.8553	0.0427	0.2507	0.0359
**Figure 4A ETS**	**One-way ANOVA test**				
	Dunnett's multiple comparisons test	Summary	Adjusted P Value	ANOVA summary	F-value
	PTZ vs. VHC	****	<0.0001		7.514
	PTZ vs. Low (ETS)	**	0.0068		
	PTZ vs. Middle (ETS)	***	0.0005		
	PTZ vs. High (ETS)	***	0.0004		
	**Tests of Normality**				
	Group	Kolmogorov-Smirnov test		Shapiro-Wilk test	
		Statistic	Sig.	Statistic	Sig.
	VHC	ns	ns	1	<0.0001
	PTZ	0.9008	0.2937	0.2039	>0.1000
	Low (ETS)	0.9564	0.744	0.1545	>0.1000
	Middle (ETS)	0.8029	0.0103	0.2221	>0.1000
	High (ETS)	0.7935	0.0121	0.2728	0.0335
	**One-way ANOVA test**				

**Figure 4B ETS**	Dunnett's multiple comparisons test	Summary	Adjusted P Value	ANOVA summary	F-value
	PTZ vs. VHC	****	<0.0001		14.17
	PTZ vs. Low (ETS)	****	<0.0001		
	PTZ vs. Middle (ETS)	****	<0.0001		
	PTZ vs. High (ETS)	****	<0.0001		
	**Tests of Normality**				
	Group	Kolmogorov-Smirnov test		Shapiro-Wilk test	
		Statistic	Sig.	Statistic	Sig.
	VHC	ns	ns	1	<0.0001
	PTZ	0.899	0.2831	0.1998	>0.1000
	Low (ETS)	0.9035	0.2393	0.2104	>0.1000
	Middle (ETS)	0.7951	0.0081	0.2906	0.01
	High (ETS)	0.7418	0.0028	0.3058	0.0086
**Figure 4C ETS**	**One-way ANOVA test**				
	Dunnett's multiple comparisons test	Summary	Adjusted P Value	ANOVA summary	F-value
	PTZ vs. VHC	****	<0.0001		12.83
	PTZ vs. Low (ETS)	***	0.0005		
	PTZ vs. Middle (ETS)	****	<0.0001		
	PTZ vs. High (ETS)	****	<0.0001		
	**Tests of Normality**				
	Group	Kolmogorov-Smirnov test		Shapiro-Wilk test	
		Statistic	Sig.	Statistic	Sig.
	VHC	ns	ns	1	<0.0001
	PTZ	0.9827	0.975	0.1204	>0.1000
	Low (ETS)	0.9715	0.9044	0.1374	>0.1000
	Middle (ETS)	0.7752	0.0044	0.3197	0.0025
	High (ETS)	0.7808	0.0085	0.3045	0.0092
**Figure 4A OXC**	**One-way ANOVA test**				
	Dunnett's multiple comparisons test	Summary	Adjusted P Value	ANOVA summary	F-value
	PTZ vs. VHC	**	0.0098		3.554
	PTZ vs. Low (OXC)	ns	0.6609		
	PTZ vs. Middle (OXC)	ns	0.4591		
	PTZ vs. High (OXC)	ns	0.9999		
	**Tests of Normality**				
	Group	Kolmogorov-Smirnov test		Shapiro-Wilk test	

		Statistic	Sig.	Statistic	Sig.
	VHC	ns	ns	1	<0.0001
	PTZ	0.9008	0.2937	0.2039	>0.1000
	Low (OXC)	0.8833	0.1699	0.1705	>0.1000
	Middle (OXC)	0.8077	0.0119	0.2919	0.0094
	High (OXC)	0.9579	0.7531	0.1493	>0.1000
**Figure 4B OXC**	**One-way ANOVA test**				
	Dunnett's multiple comparisons test	Summary	Adjusted P Value	ANOVA summary	F-value
	PTZ vs. VHC	****	<0.0001		6.968
	PTZ vs. Low (OXC)	**	0.0011		
	PTZ vs. Middle (OXC)	**	0.0013		
	PTZ vs. High (OXC)	**	0.008		
	**Tests of Normality**				
	Group	Kolmogorov-Smirnov test		Shapiro-Wilk test	
		Statistic	Sig.	Statistic	Sig.
	VHC	ns	ns	1	<0.0001
	PTZ	0.899	0.2831	0.1998	>0.1000
	Low (OXC)	0.9348	0.5281	0.2062	>0.1000
	Middle (OXC)	0.7601	0.0028	0.2652	0.0295
	High (OXC)	0.9371	0.461	0.1654	>0.1000
**Figure 4C OXC**	**One-way ANOVA test**				
	Dunnett's multiple comparisons test	Summary	Adjusted P Value	ANOVA summary	F-value
	PTZ vs. VHC	***	0.0001		5.941
	PTZ vs. Low (OXC)	*	0.0121		
	PTZ vs. Middle (OXC)	**	0.0086		
	PTZ vs. High (OXC)	ns	0.0775		
	**Tests of Normality**				
	Group	Kolmogorov-Smirnov test		Shapiro-Wilk test	
		Statistic	Sig.	Statistic	Sig.
	VHC	ns	ns	1	<0.0001
	PTZ	0.9827	0.975	0.1204	>0.1000
	Low (OXC)	0.9089	0.3085	0.1706	>0.1000
	Middle (OXC)	0.9268	0.3797	0.1569	>0.1000
	High (OXC)	0.7708	0.0044	0.278	0.011
	**One-way ANOVA test**				

**Figure 4A TGB**	Dunnett's multiple comparisons test	Summary	Adjusted P Value	ANOVA summary	F-value
	PTZ vs. VHC	*	0.0289		2.978
	PTZ vs. Low (TGB)	ns	0.9961		
	PTZ vs. Middle (TGB)	ns	0.4841		
	PTZ vs. High (TGB)	ns	0.1846		
	**Tests of Normality**				
	Group	Kolmogorov-Smirnov test		Shapiro-Wilk test	
		Statistic	Sig.	Statistic	Sig.
	VHC	ns	ns	1	<0.0001
	PTZ	0.9008	0.2937	0.2039	>0.1000
	Low (TGB)	0.8925	0.127	0.2231	>0.1000
	Middle (TGB)	0.8775	0.0968	0.232	>0.1000
	High (TGB)	0.808	0.0116	0.2061	>0.1000
**Figure 4B TGB**	**One-way ANOVA test**				
	Dunnett's multiple comparisons test	Summary	Adjusted P Value	ANOVA summary	F-value
	PTZ vs. VHC	****	<0.0001		8.337
	PTZ vs. Low (TGB)	**	0.0023		
	PTZ vs. Middle (TGB)	***	0.0002		
	PTZ vs. High (TGB)	****	<0.0001		
	**Tests of Normality**				
	Group	Kolmogorov-Smirnov test		Shapiro-Wilk test	
		Statistic	Sig.	Statistic	Sig.
	VHC	ns	ns	1	<0.0001
	PTZ	0.899	0.2831	0.1998	>0.1000
	Low (TGB)	0.9141	0.2405	0.1773	>0.1000
	Middle (TGB)	0.912	0.2574	0.1441	>0.1000
	High (TGB)	0.8616	0.0511	0.2571	0.0276
**Figure 4C TGB**	**One-way ANOVA test**				
	Dunnett's multiple comparisons test	Summary	Adjusted P Value	ANOVA summary	F-value
	PTZ vs. VHC	****	<0.0001		7.138
	PTZ vs. Low (TGB)	**	0.002		
	PTZ vs. Middle (TGB)	**	0.0026		
	PTZ vs. High (TGB)	**	0.0014		
	**Tests of Normality**				
	Group	Kolmogorov-Smirnov test		Shapiro-Wilk test	

		Statistic	Sig.	Statistic	Sig.
	VHC	ns	ns	1	<0.0001
	PTZ	0.9827	0.975	0.1204	>0.1000
	Low (TGB)	0.9458	0.5762	0.1893	>0.1000
	Middle (TGB)	0.8661	0.0691	0.2065	>0.1000
	High (TGB)	0.8768	0.0798	0.1781	>0.1000
**Figure 4A TOP**	**One-way ANOVA test**				
	Dunnett's multiple comparisons test	Summary	Adjusted P Value	ANOVA summary	F-value
	PTZ vs. VHC	***	0.0001		6.309
	PTZ vs. Low (TOP)	*	0.0124		
	PTZ vs. Middle (TOP)	**	0.0059		
	PTZ vs. High (TOP)	**	0.0027		
	**Tests of Normality**				
	Group	Kolmogorov-Smirnov test		Shapiro-Wilk test	
		Statistic	Sig.	Statistic	Sig.
	VHC	0.9777	0.9509	0.1546	>0.1000
	PTZ	0.9463	0.6493	0.1847	>0.1000
	Low (TOP)	0.913	0.3373	0.1656	>0.1000
	Middle (TOP)	0.9806	0.9659	0.1435	>0.1000
	High (TOP)	0.9231	0.3834	0.1805	>0.1000
**Figure 4B TOP**	**One-way ANOVA test**				
	Dunnett's multiple comparisons test	Summary	Adjusted P Value	ANOVA summary	F-value
	PTZ vs. VHC	****	<0.0001		11.06
	PTZ vs. Low (TOP)	***	0.0004		
	PTZ vs. Middle (TOP)	***	0.0006		
	PTZ vs. High (TOP)	****	<0.0001		
	**Tests of Normality**				
	Group	Kolmogorov-Smirnov test		Shapiro-Wilk test	
		Statistic	Sig.	Statistic	Sig.
	VHC	0.8769	0.1759	0.1814	>0.1000
	PTZ	0.9605	0.8037	0.2036	>0.1000
	Low (TOP)	0.9231	0.4182	0.1767	>0.1000
	Middle (TOP)	0.8072	0.0342	0.2387	>0.1000
	High (TOP)	0.9293	0.4413	0.1867	>0.1000
	**One-way ANOVA test**				

	Dunnett's multiple comparisons test	Summary	Adjusted P Value		F-value
**Figure 4C TOP**	PTZ vs. VHC	ns	0.0611	ANOVA summary	2.746
	PTZ vs. Low (TOP)	ns	0.2355		
	PTZ vs. Middle (TOP)	ns	0.9023		
	PTZ vs. High (TOP)	*	0.0278		
	**Tests of Normality**				
	Group	Kolmogorov-Smirnov test		Shapiro-Wilk test	
		Statistic	Sig.	Statistic	Sig.
	VHC	0.8622	0.1262	0.2038	>0.1000
	PTZ	0.8558	0.0863	0.2581	0.0856
	Low (TOP)	0.892	0.209	0.2033	>0.1000
	Middle (TOP)	0.8475	0.0899	0.218	>0.1000
	High (TOP)	0.9321	0.469	0.2092	>0.1000
